# TSLP induces a proinflammatory phenotype in circulating innate cells and predicts prognosis in sepsis patients

**DOI:** 10.1002/2211-5463.12746

**Published:** 2019-12-02

**Authors:** Qichuan Yu, Yang Li, Hao Wang, Huawei Xiong

**Affiliations:** ^1^ Department of Orthopedics The First Affiliated Hospital of Nanchang University China; ^2^ Department of Emergency The First Affiliated Hospital of Nanchang University China

**Keywords:** cytokine, proinflammatory phenotype, sepsis, thymic stromal lymphopoietin, TSLP

## Abstract

Thymic stromal lymphopoietin (TSLP) has been identified as a crucial inflammatory cytokine in immune homeostasis. Previous studies have reported conflicting effects of TSLP on sepsis in mice, and the effect of TSLP on sepsis in humans has not been investigated. In this study, we used the ELISA to measure serum levels of TSLP in patients with sepsis, and used flow cytometry and ELISA to evaluate the proinflammatory phenotype of circulating immune cells. In addition, we used quantitative RT‐PCR to examine the expression of proinflammatory cytokines [interleukin (IL)‐1β, IL‐6, tumor necrosis factor‐α, transferrin growth factor‐β, IL‐10, and matrix metalloproteinase] between patients with high and low levels of TSLP. Flow cytometry analysis was performed to evaluate the phagocytic and respiratory burst of circulating neutrophils. A significant increase in the production of proinflammatory cytokines by nonclassical monocytes and the number of interferon (IFN)‐γ^+^ CD4^+^ monocytes was observed in patients with high levels of TSLP. Furthermore, the number of IL‐10^+^ regulatory T cells was observed to be increased in patients with high levels of TSLP. We found that TSLP values greater than 350 pg·mL^−1^ were associated with a higher mortality rate and longer stays in intensive care (sensitivity of 89% and specificity of 79%). In patients with low levels of neutrophils, the area under curve was only 0.71 (based on the cutoff value in the diagnostic test evaluation; sensitivity of 62% and specificity of 68%). Our findings suggest that the serum levels of TSLP may be suitable as a biomarker for prediction of prognosis in a subgroup of patients with sepsis who are exhibiting hyperleukocytosis and a high neutrophil ratio.

## Abstract 2

Elevated serum levels of thymic stromal lymphopoietin lead to an increase in the production of proinflammatory cytokines in CD14^+^CD16^2+^ monocytes, CD14^+^CD16^+^ monocytes and CD4^+^ T cells, which might be correlated with higher mortality of patients with sepsis with hyperleukocytosis or high neutrophil ratio.

## Graphical abstract

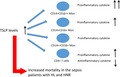

**Abbreviations**
AUC: area under curve
CLP: cecal ligation and puncture
DC: dendritic cell
FITC: fluorescein isothiocyanate
Foxp3: forkhead box P3
HKSA: heat‐killed *Staphylococcus aureus*
HL: hyperleukocytosis
HNR: high neutrophil ratio
ICU: intensive care unit
IFN: interferon
IL: interleukin
PBMC: peripheral blood mononuclear cell
SD: standard deviation
SIRS: systemic inflammation response syndrome
Th: T helper
TNF: tumor necrosis factor
Treg: regulatory T
TSLP: thymic stromal lymphopoietin
TSLPR: TSLP receptor

Thymic stromal lymphopoietin (TSLP) has been identified as a crucial inflammatory cytokine in immune homeostasis. TSLP is known to trigger dendritic cells (DCs) and promote differentiation of T helper (Th) type 2 cells, and is linked to the pathogenesis of some allergic diseases, including asthma and atopic dermatitis 1, 2. In addition, TSLP may influence the activation of regulatory T (Treg) and Th2 cells by suppressing Th1 cells and exert an anti‐inflammatory effect on colitis and atherosclerotic plaque 3, 4. The extension of atherosclerostic plaque was observed in apoE^−/−^ mice. Dextran sodium sulfate–induced colitis was more severe in TSLP receptor (TSLPR)^−/−^ mouse than in wild‐type mouse. The ambivalent effect of TSLP on different tissues is unclear, and further investigations in humans revealed two isoforms 5, 6, including the longer isoform and shorter isoform. The shorter isoform is constitutively expressed and mediates immune homeostasis, whereas the longer isoform is expressed at a very low level in a steady state and is upregulated in the inflammatory microenvironment 7. These studies demonstrate the complex biological function of TSLP.

Previous studies about the interrelationship between TSLP and sepsis have revealed the elevated levels of TSLP in a colorectal ligation and puncture animal model. However, the effect of TSLP in sepsis mice was opposite in two studies on a cecal ligation and puncture (CLP) mouse model. In the first study, the administration of a neutralizing antibody to TSLP in mice with sepsis resulted in decreased mortality and weakened the host immune response 8. In the second study, CLP‐induced sepsis caused higher mortality and pejorative inflammation but eliminated the bacterial infection in TSLP^−/−^ mouse 9. These inconsistent results suggest that TSLP could play various roles in the regulation of different immune cells. However, no clinical investigation has explored the effect and potential value of TSLP in patients with sepsis. Studies are warranted to evaluate whether the serum levels of TSLP are increased in patients with sepsis or if higher TSLP levels predict improved prognosis.

In this study, we demonstrate elevated levels of TSLP in patients with sepsis and reveal the negative correlation between serum levels of TSLP in patients with sepsis with a high ratio of neutrophils and mortality. The findings of this study reveal the insufficient infiltration of neutrophils in infectious lesions, as observed in animal experiments. Furthermore, we show that TSLP mainly stimulated the activation of monocytes and induced their proinflammatory phenotype rather than T lymphocytes and neutrophils among the circulating immune cells. These findings suggest that TSLP may serve as a potential biomarker for the prediction of mortality in a subgroup of patients with sepsis, particularly in patients with a high ratio of neutrophils.

## Materials and methods

### Patient enrollment

Patient enrollment was performed from 2016 to 2018 in our institution. All patients were diagnosed with sepsis. The sepsis was identified according to two or more systemic inflammation response syndrome (SIRS) criteria, including changes in white blood cell count, temperature, respiratory rate and heart rate reflecting inflammation, in combination with life‐threatening organ dysfunction based on the Third Consensus Definition for Sepsis and Septic Shock (Sepsis‐3) 10. A total of 228 patients were enrolled. Routine blood examination revealed that 134 patients had hyperleukocytosis (HL) and high neutrophil ratio (HNR). Serum samples from these patients were isolated, and the levels of TSLP were measured with an ELISA. HL was defined as leukocyte number greater than 11.5 × 10^9^ L^−1^, and HNR was defined as the percentage of neutrophils greater than 75%. The patients with a high level of TSLP were defined as more than the average level of TSLP in the corresponding patient subset. The patients with a low level of TSLP were defined as less than the average level of TSLP in the corresponding patient subset. The inclusion criteria were as follows: (a) age between 20 and 60 years; (b) no severe cardiopulmonary failure during serum collection, including mechanical ventilation, support of extracorporeal membrane oxygenation and administration of vasoactive drug for maintenance of blood pressure; (c) procalcitonin > 0.5 μg·L^−1^; and (d) no neoplastic, allergic and autoimmune diseases. The exclusion criteria included occurrence of cardiac arrest, possibility or definite diagnosis of viral and other atypical pathogenic infections, pregnant women and use of immunosuppressants and glucocorticoids. We then measured the functional characterization and frequency of inflammatory cells in all patients who suffered from sepsis regardless of the number of leukocytes and ratio of neutrophils, and compared the difference between patients with higher and lower levels of TSLP in the functional characterization and frequency of inflammatory cells. The study conformed to the Declaration of Helsinki and was approved by the medical ethics committee of the First Affiliated Hospital of Nanchang University. All patients enrolled in this study signed informed consent forms.

### ELISA

Blood samples from patients were collected, and serum was isolated for the measurement of TSLP levels. Serum TSLP levels were measured according to ELISA method using a human TSLP ELISA detection kit (DY1398; R&D Systems, Minneapolis, MN, USA). All samples were measured in duplicates, and the mean concentration was calculated. ELISA was also used for the measurement of cytokine levels in the supernatant fluid collected from the cultured peripheral blood mononuclear cells (PBMCs) subjected to stimulation with lipopolysaccharide (2 μL·mL^−1^ of cell culture fluid). All ELISA detection kits were purchased from Abcam Co. (Cambridge, UK) for the measurement of cytokines secreted from PBMCs isolated from patients, including the human interleukin (IL)‐1β ELISA Kit (ab46608, Abcam.Co), human IL‐6 ELISA Kit (ab46027, Abcam.Co), human matrix metalloproteinase‐9 ELISA Kit (ab100610, Abcam.Co), human tumor necrosis factor (TNF)‐α ELISA Kit (ab181421) and human IL‐10 ELISA Kit (ab100519,Abcam.Co).

### Flow cytometry analysis of phagocytic and respiratory burst

The flow cytometry was used for the analysis of phagocytosis and respiratory burst of neutrophils as described previously 11. Neutrophils from whole blood samples were adjusted to a cell density of 3 × 10^6^ cells·mL^−1^, followed by *in vitro* coculture with fluorescein isothiocyanate (FITC)‐labeled bacteria [heat‐killed *Staphylococcus aureus* (HKSA)] at multiplicity of infection of 10 : 1 for 60 min or with PMA (800 ng·mL^−1^) for 15 min at 37 °C. This was followed by the addition of 25 mL of a 2800 ng·mL^−1^ solution of hydroethidine (Sigma, San Francisco, CA, USA) and incubation for 5 min at 37 °C. Erythrocytes were excluded with lysis buffer (BD Biosciences, San Jose, CA, USA) before analysis by flow cytometry (FACSCalibur; BD Biosciences). The number of neutrophils with phagocytic function in 1‐mL samples of whole blood was calculated by multiplying absolute neutrophil counts (determined using automated blood counter) with the percentage of phagocytosis (analyzed by flow cytometry) and dividing the obtained value by 100. Respiratory burst function, which occurs after phagocytosis, was subsequently calculated by multiplying the number of phagocytosing neutrophils (calculated earlier) by the percentage of respiratory burst (analyzed by flow cytometry) and dividing the obtained value by 100.

### Flow cytometry analysis of intracellular cytokine staining

We obtained PBMCs from patients with high or low levels of TSLP by density centrifugation using Ficoll‐Paque and cultured these cells in RPMI 1640 supplemented with 10% FBS. Cells were resuspended at a density of 0.5 × 10^5^ cells·mL^−1^, and 100–200 μL of cell suspension was stimulated with cell stimulation cocktail (plus protein transport inhibitors; 1.5 μL·mL^−1^ of cell suspension; BD Biosciences). After stimulation *in vitro*, these cells were permeabilized with fixation and permeabilization buffer kit (BD Biosciences) and stained for 30 min at 4 °C in flow tubes with directly conjugated antibodies. The cells were fixed with 4% paraformaldehyde. The antibodies used for the flow cytometry analysis included anti‐human CD4‐allophycocyanin (560158; BD Biosciences), anti‐human IFN‐γ‐PeCy7 (557844; BD Biosciences), anti‐human IL‐4 (554516; BD Biosciences), anti‐human forkhead box P3 (Foxp3)‐Alexa Fluor 488 (560459; BD Biosciences) and anti‐human IL‐10‐phycoerythrin (554498; BD Biosciences). The cells were separated with a multicolor flow cytometer (BD Company, Franklin lakes, NJ, USA), and the fluorescence‐activated cell signaling data were analyzed by flowjo 10.0 software (BD Bioscience, San Jose, CA, USA).

### Quantitative RT‐PCR

Total RNA was purified from cells using RNeasy Kit (Qiagen, Hilden, Germany). cDNA synthesis was performed using SuperScript III reverse transcriptase (Invitrogen, Carlsbad, CA, USA) and random hexamers. Real‐time PCRs were carried out using the SYBR Green PCR kit and the primers listed in Table 1 on the Applied Biosystems 7500 Fast Real‐Time PCR System (Applied Biosystems, Carlsbad, CA, USA). Expression levels of genes were normalized to the expression level of glyceraldehyde‐3‐phosphate dehydrogenase. Results were quantified using the $2^{-\Delta \Delta C_{t}}$ method.

**Table 1:** Primers for quantitative RT‐PCR.

Gene	Upstream primer	Downstream primer
*IL‐1β*	5′‐GACCCTAGGCCATG‐3′	5′‐TACCCGGCACACTT‐3′
*TNF‐α*	5′‐GTACGACTTGGTAG‐3′	5′‐AGCCGTGGTTACCA‐3′
*IFN‐γ*	5′‐CCGTGGACCAACTG‐3′	5′‐GTGATGATGGTACA‐3′
*IL‐6*	5′‐CACGATACCCAAGTA‐3′	5′‐GTGACCCATCAGCCA‐3′

### Data analysis

Data were entered into an Excel spreadsheet (Microsoft Corporation, Redmond, WA, USA). Analysis was conducted in Excel and spss statistics 20 (IBM Corporation, Armonk, NY, USA). Sensitivity, specificity, positive predictive value and negative predictive value were calculated, as described by the TDR Diagnostics Evaluation Expert Panel. Patients were stratified according to the serum levels of TSLP (lower level: less than mean; higher level: greater than or equal to mean) for *ex vivo* investigation. Chi‐square test was used in comparison of two‐category data between patients with or without HL and HNR. Fisher’s exact test was used when the smallest expected count is less than 5 in the data instead of classical chi‐square test. The area under curve (AUC) was calculated in patients with or without HL and HNR according to the cutoff value. The sensitivity, specificity, positive predictive value and negative predictive value were calculated according to the best cutoff value in the diagnosis evaluation test. The corresponding best cutoff value used for AUC value and diagnostic evaluation test was determined according the Youden index (specificity + sensitivity − 1). The largest Youden index indicated that the corresponding cutoff value is the best.

Other quantitative data were represented by means ± standard deviation (SD). Statistical significance was determined by the Student’s *t*‐test using spss statistics 20: *P < 0.05, **P < 0.01, ***P < 0.001.

## Results

### Patients baseline characteristics and clinical outcomes

**Figure 1:**
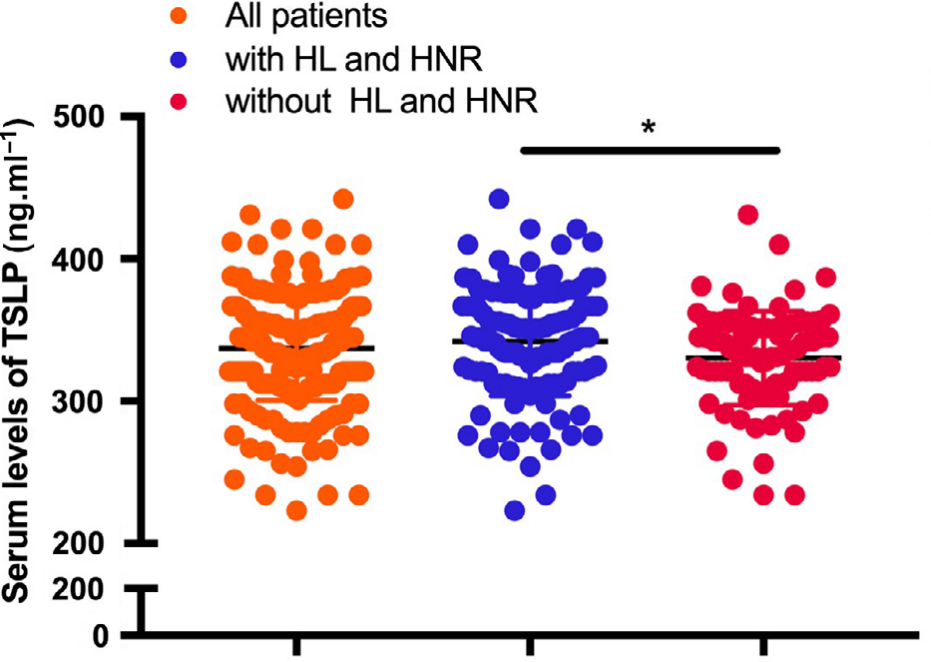
Scatterplot about the serum levels of TSLP in patients with sepsis. The levels of TSLP in the patients with HL and HNR were significantly higher than in those without HL and HNR. The unpaired Student’s *t*‐test was used for statistical analysis. The two‐sided test was applied. *P* < 0.05 indicated statistical significance: *P < 0.05. *n* = 10.

The average value of TSLP was 337.1 pg·mL^−1^ in the total population. The average value of TSLP was 341.9 pg·mL^−1^ in those patients with HL and HNR. The average value of TSLP was 330.3 pg·mL^−1^ in patients without HL and HNR. The baseline characteristics are presented in Table 2. The levels of TSLP in the patients with HL and HNR were significantly higher than in those without HL and HNR, which might be because of the role of TSLP in the mobilization of leukocytes and neutrophils (Fig. 1). Considering the influence of neutrophil infiltration on bacterial killing and the fact that the levels of TSLP have no effect on the function of neutrophils, we compared the in‐hospital mortality between different subsets (with or without HL and high ratio of neutrophils) according to the stratification of TSLP levels. As a result, we observed higher mortality rates in patients with high levels of TSLP accompanied with HL and HNR than in those with low levels of TSLP (Table 2). In contrast, the serum TSLP level had no influence on the mortality of patients without HL and HNR (Table 3). Moreover, patients with high TSLP levels had longer stays in the intensive care unit (ICU) than those with low TSLP levels. TSLP levels had no effect on ICU stay in patients without HL and HNR. Thus, TSLP may exert adverse effects on the clinical outcomes in patients with sepsis with an intense immune response, which may lead to secondary systemic inflammatory response syndrome and secondary multiple organ failure.

**Table 2:** Patient baseline characteristics in the group with HL and high ratio of neutrophils (*n* = 134). APACHE, Acute Physiologic Assessment and Chronic Health Evaluation.

Parameters	High level (*n* = 87)	Low level (*n* = 47)	*P*‐value
Age (years), mean ± SD	62.1 ± 17.4	59.7 ± 12.8	0.248
Male, *n* (%)	40 (45.9)	27 (57.4)	0.131
Neutrophils (%), mean ± SD	85.2 ± 4.1	81.3 ± 2.6	0.043
Leukocytes (×10^9^), mean ± SD	15.6 ± 2.2	16.0 ± 2.6	0.141
TSLP levels (pg·mL^−1^), mean ± SD	425.6 ± 52.4	305.6 ± 21.6	0.004
C‐reactive protein (mg·L^−1^), mean ± SD	8.3 ± 1.1	7.7 ± 1.4	0.052
Procalcitonin, mean ± SD	2.81 ± 0.47	2.03 ± 0.34	0.031
Random blood glucose (mmol·L^−1^), mean ± SD	9.4 ± 1.2	9.7 ± 1.7	0.067
Organ dysfunction, *n* (%)			
Acute respiratory syndrome	53 (60.9)	17 (36.1)	0.012
Renal failure	40 (45.9)	15 (31.9)	0.057
Heart failure	10 (11.5)	7 (14.9)	0.128
Multiple organ dysfunction, *n* (%)	36 (41.3)	9 (19.1)	0.045
Septic shock, *n* (%)	22 (25.2)	7 (14.8)	0.072
Heart rate, mean ± SD	102.3 ± 16.7	98.7 ± 21.5	0.081
Respiratory rate, mean ± SD	25.7 ± 6.8	23.3 ± 5.9	0.140
Blood pressure (mmHg), mean ± SD			
Systolic	121.8 ± 27.6	126.5 ± 31.6	0.081
Diastolic	77.1 ± 21.1	82.3 ± 17.8	0.096
APACHE II score, mean ± SD	25.1 ± 4.1	20.2 ± 3.7	0.017
In‐hospital mortality, *n* (%)	31 (35.6)	6 (12.7)	0.021
Length of stay in ICU (days), mean ± SD	9.7 ± 2.1	7.5 ± 1.0	0.015
Comorbidity, *n* (%)			
Chronic obstructive pulmonary disease	46 (52.8)	30 (53.1)	0.380
Diabetes	23 (26.4)	10 (21.2)	0.313
Coronary artery disease	16 (18.3)	7 (14.9)	0.265
Cancer	5 (5.7)	3 (6.3)	0.246

**Table 3:** Patient baseline characteristics in the group without HL and high ratio of neutrophils (*n* = 94). APACHE, Acute Physiologic Assessment and Chronic Health Evaluation.

Parameter	High level (*n* = 52)	Low level (*n* = 42)	*P*‐value
Age (years), mean ± SD	58.5 ± 12.9	60.6 ± 15.7	0.167
Male, *n* (%)	30 (57.6)	21 (50)	0.221
Neutrophils (%), mean ± SD	60.2 ± 8.7	61.3 ± 6.3	0.175
Leukocytes (×10^9^), mean ± SD	8.6 ± 1.7	8.4 ± 1.1	0.141
TSLP levels (pg·mL^−1^), mean ± SD	397.6 ± 33.4	276.0 ± 27.9	0.002
C‐reactive protein (mg·L^−1^), mean ± SD	4.8 ± 2.1	4.7 ± 2.8	0.125
Procalcitonin, mean ± SD	1.69 ± 0.87	1.78 ± 0.71	0.121
Random blood glucose (mmol·L^−1^), mean ± SD	8.7 ± 1.7	7.9 ± 1.9	0.057
Organ dysfunction, *n* (%)			
Acute respiratory syndrome	32 (61.5)	26 (61.9)	0.314
Renal failure	25 (48.1)	17 (40.5)	0.198
Heart failure	16 (30.8)	14 (33.3)	0.271
Multiple organ dysfunction, *n* (%)	24 (46.1)	16 (38.0)	0.145
Septic shock, *n* (%)	17 (32.6)	13 (30.9)	0.255
Heart rate, mean ± SD	108.3 ± 27.6	106.9 ± 25.8	0.183
Respiratory rate, mean ± SD	26.5 ± 4.8	25.8 ± 4.5	0.211
Blood pressure (mmHg), mean ± SD			
Systolic	126.3 ± 23.4	128.6 ± 25.5	0.176
Diastolic	84.6 ± 21.1	86.9 ± 19.9	0.189
APACHE II score, mean ± SD	22.1 ± 5.8	21.6 ± 4.7	0.103
In‐hospital mortality, *n* (%)	20 (38.4)	16 (38.0)	0.271
Length of stay in ICU (days), mean ± SD	10.7 ± 1.1	10.3 ± 1.0	0.296
Comorbidity, *n* (%)			
Chronic obstructive pulmonary disease	24 (46.1)	18 (42.8)	0.270
Diabetes	13 (25.0)	8 (19.0)	0.171
Coronary artery disease	17 (32.7)	11 (26.1)	0.163
Cancer	5 (9.6)	6 (14.3)	0.127

### Patients with sepsis with high levels of TSLP show obvious proinflammatory phenotype for circulating CD14^+^CD16^+^ monocytes

**Figure 2:**
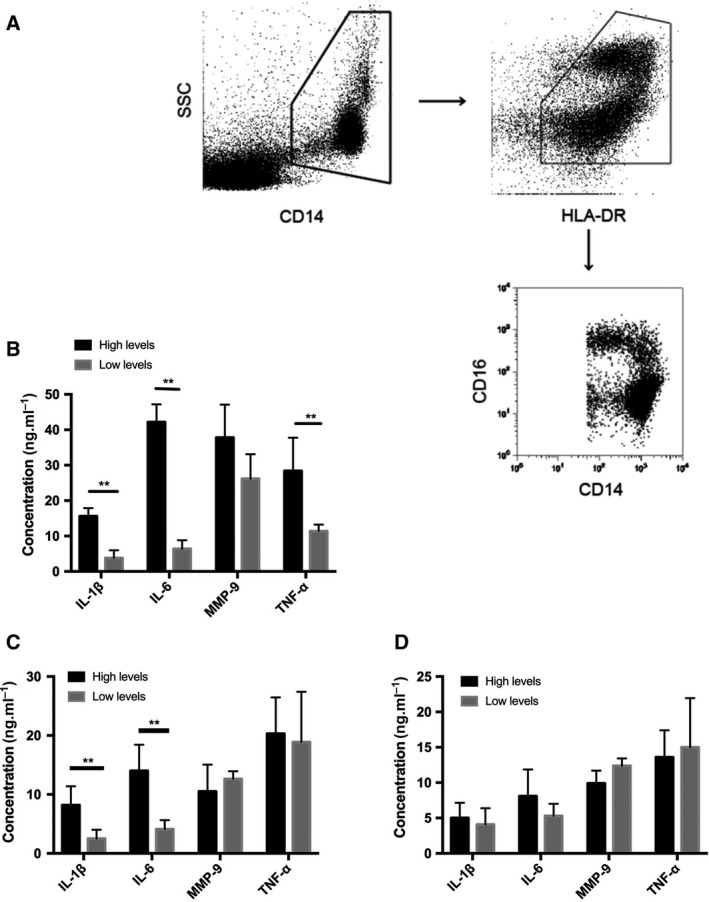
The levels of cytokines producing by different subtypes of monocyte from PBMCs of patients *in vitro*. (A) The sorting strategy for the subtypes of monocytes from PBMCs of patients with sepsis. The CD14^2+^CD16^−^, CD14^+^CD16^+^ intermediate and CD14^low^CD16^2+^ monocytes were isolated from CD16^+^CD14^+^ monocytes according to flow sorting. (B) The levels of inflammatory cytokines producing by PBMCs from patients with a higher or lower level of TSLP in cultural supernatant after stimulation with lipopolysaccharide (LPS). (C) The levels of inflammatory cytokines producing by CD14^low^CD16^2+^ nonclassical monocytes from patients with a higher or lower level of TSLP in cultural supernatant after stimulation with LPS. (D) The levels of inflammatory cytokines producing by CD14^2+^CD16^−^ nonclassical monocytes from patients with a higher or lower level of TSLP in cultural supernatant after stimulation with LPS. Data are presented by mean ± SD (error bar). The unpaired Student’s *t*‐test was used for statistical analysis. The two‐sided test was applied. *P* < 0.05 indicated statistical significance: **P < 0.01. *n* = 10. HLA, human leukocyte antigen.

**Figure 3:**
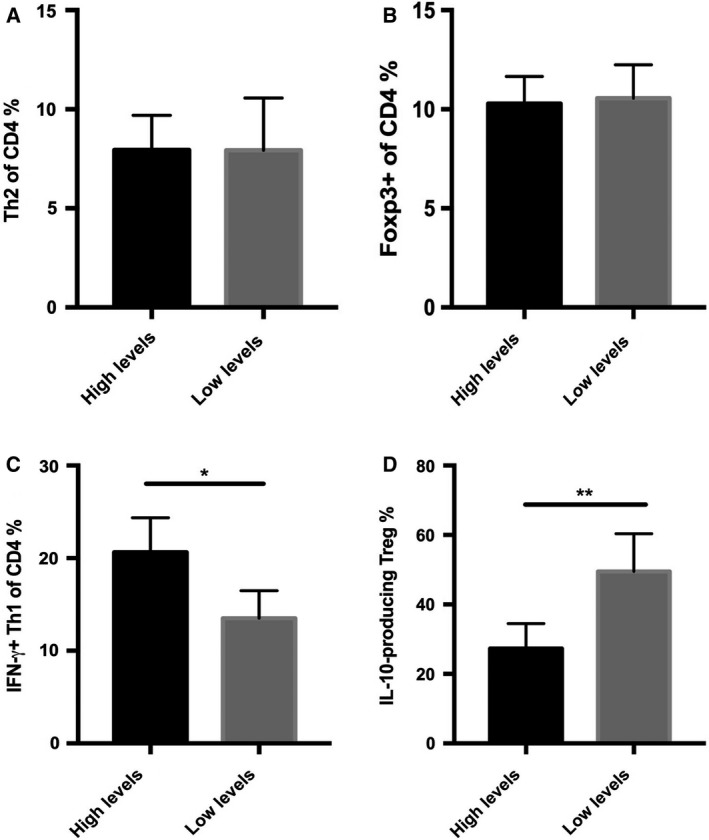
The frequency of Th1, Th2 and IL‐10^+^ Treg cells in the CD4^+^ T cells from patients. (A) The frequency of IL‐4^+^ Th2 measured by intracellular flow cytometry assay from PBMCs of patients with a higher or lower level of TSLP. (B) The frequency of CD4^+^CD25^+ ^Foxp3^+^ Treg cells measured by flow cytometry from PBMCs of patients with higher or lower levels of TSLP. (C) The frequency of proinflammatory IFN‐γ^+^ Th1 cells measured by flow cytometry from PBMCs of patients with higher or lower levels of TSLP. (D) The frequency of proinflammatory IFN‐γ^+^ Th1 measured by flow cytometry from PBMCs of patients with higher or lower levels of TSLP. Data are presented by mean ± SD (error bar). The unpaired Student’s *t*‐test was used for statistical analysis. The two‐sided test was applied. *P* < 0.05 indicated statistical significance: *P < 0.05, **P < 0.01. *n* = 10.

The protein TSLP acts as an inflammatory cytokine and exerts specific effects on circulating immune cells from patients with sepsis with high TSLP levels. Therefore, we evaluated the inflammatory phenotype of the circulating immune cells by flow cytometry and ELISA in patients with sepsis. Figure 2A indicates the sorting strategy for the further analysis. We found that CD14^+^CD16^+^ (intermediate) and CD14^−^CD16^2+^ (nonclassical) monocytes from patients with sepsis with high levels of TSLP (over the mean value) produced significantly increased levels of inflammatory cytokines, including IL‐1β, IL‐6, matrix metalloproteinase‐9 and TNF‐α, as compared with those isolated from patients with low TSLP levels (Fig. 2B,C). However, no difference was observed in the levels of cytokines produced by the classical CD14^2+^CD16^−^ monocytes from patients with high and low TSLP levels (Fig. 2D). Furthermore, no difference was observed in the number of IL‐4^+^ Th2 and Foxp3^+^ Treg cells in CD4^+^ T lymphocytes obtained from the two groups of patients (Fig. 3A,B). However, patients with high levels of TSLP showed a significant increase in the number of Th1 cells, as well as a decrease in the ratio of IL‐10^+^ Treg to total Treg cells (Fig. 3C,D). These results demonstrate that TSLP aggravated the inflammatory response in patients with sepsis, as evident from the increase in the frequency of proinflammatory immune cells and production of inflammatory cytokines.

### TSLP has no effect on the phagocytic and respiratory burst of circulating neutrophils in patients with high levels of TSLP

**Figure 4:**
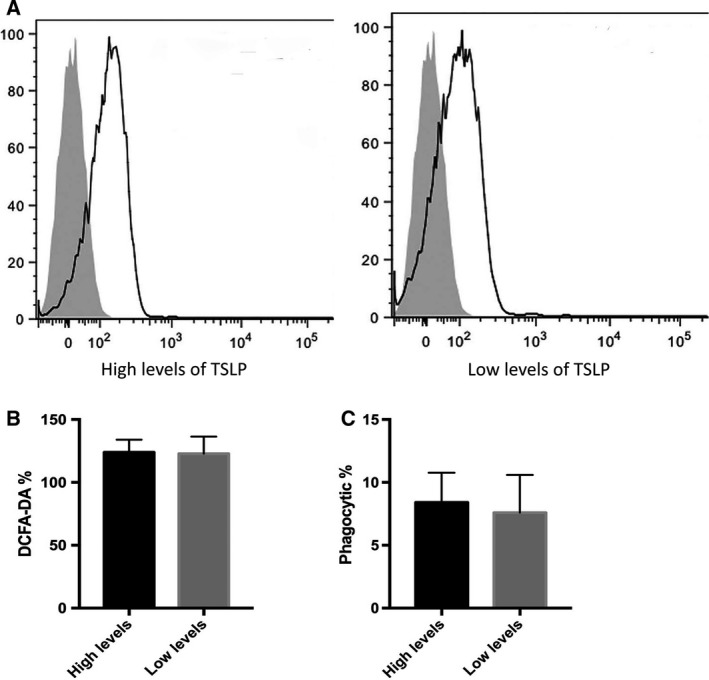
The evaluation of phagocytosis and production of reactive oxygen species in the neutrophils isolated from patients. (A) The representative figures of mean fluorescent intensity (MFI) evaluated by flow cytometry in the neutrophils stimulated by HKSA*.* (B) The quantitative analysis of 2’,7’‐dichlorodihydrofluorescin diacetate intracellular staining in the HKSA/PMA‐stimulating neutrophils isolated from patients with a higher or lower level of TSLP. (C) The frequency of FITC‐labeled phagocytic neutrophils observed by fluorescent microscope in the neutrophils from patients. Data are presented as mean ± SD (error bar). Two‐sided Student’s *t*‐test was applied. *P* < 0.05 indicated statistical significance. *n* = 10.

To evaluate the effect of TSLP on the function of neutrophils in patients with sepsis, we performed flow cytometry analysis and explored the functional changes in neutrophils in all sepsis regardless of whether these patients suffered from HNR and HL. We found that the number of phagocytic neutrophils (Fig. 4A,B) and the percentage of respiratory burst (Fig. 4C) in neutrophils isolated from patients with high TSLP levels were similar to those observed for patients with low levels of TSLP, as observed after coculturing with FITC‐labeled bacteria (HKSA) or incubation with PMA and hydroethidine. No statistical difference was observed in the percentage of ethidium bromide–positive cells and the number of phagocytic neutrophils between patients with high and low TSLP levels. These data suggest that the elevated levels of TSLP would have no influence on the function of circulating neutrophils, including phagocytosis and production of reactive oxygen species. These results suggest that elevated levels of TSLP in patients with sepsis would induce proinflammatory phenotype in circulating mononuclear cells but may not promote the activation of polymorphonuclear cells. This change may lead to the production of lysozymes, which may play an important role in innate antimicrobial response.

### Patients with sepsis with high levels of TSLP show an increase in the expression of cytokines

**Figure 5:**
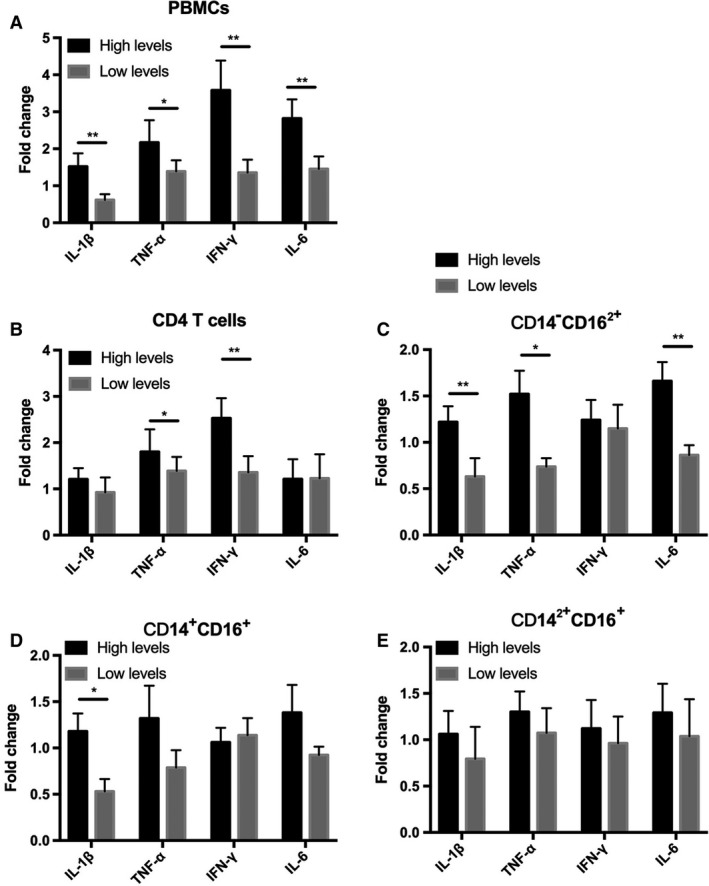
The expression of proinflammatory and anti‐inflammatory cytokines in the different subtypes of immune cell from patients. (A) The expression of cytokines in the PBMCs from patients was detected by quantitative RT‐PCR. (B) The expression of cytokines in the CD4^+^ T cells from patients. (C) The expression of cytokines in the CD14^low^CD16^2+^ monocytes from patients. (D) The expression of cytokines in the CD14^+^CD16^+^ monocytes from patients. (E) The expression of cytokines in the CD14^2+^CD16^−^ monocytes from patients. Data are presented as mean ± SD (error bar). Two‐sided Student’s *t*‐test was applied. *P* < 0.05 indicated statistical significance: *P < 0.05, **P < 0.01. *n* = 10.

We directly measured the expression of cytokines in PBMCs from patients with high or low levels of TSLP. A significant increase in the expression of IL‐1β, IFN‐γ, TNF‐α and IL‐6 was observed in patients with high TSLP levels compared with patients with low TSLP levels, regardless of the number of leukocytes and ratio of neutrophils (Fig. 5A). We isolated and purified CD14^+^CD16^+^, CD14^−^CD16^2+^ and CD14^2+^CD16^−^ monocytes from PBMCs and CD4^+^ T cells to measure the expression of proinflammatory cytokines. As a result, we found an increase in the expression of TNF‐α and IFN‐γ in CD4^+^ T cells obtained from patients with high TSLP levels (Fig. 5B). In addition, a decrease in the expression of IL‐10 was observed in CD4^+^ T cells obtained from patients with high levels of TSLP as compared with those obtained from patients with low levels of TSLP (Fig. 5B). Furthermore, an increase in the expression of IL‐1β, TNF‐α and IL‐6 was observed in CD14^+^CD16^+^ and CD14^−^CD16^2+^ monocytes obtained from patients with high levels of TSLP (Fig. 5C,D). No difference was observed in the expression level of classical monocytes between the two patient groups (Fig. 5E). These observations are consistent with the results of flow cytometry analysis and ELISA. These data indicate that TSLP may induce the preferential proinflammatory phenotype.

### Elevated serum levels of TSLP predict the survival of patients with HL and HNR

**Figure 6:**
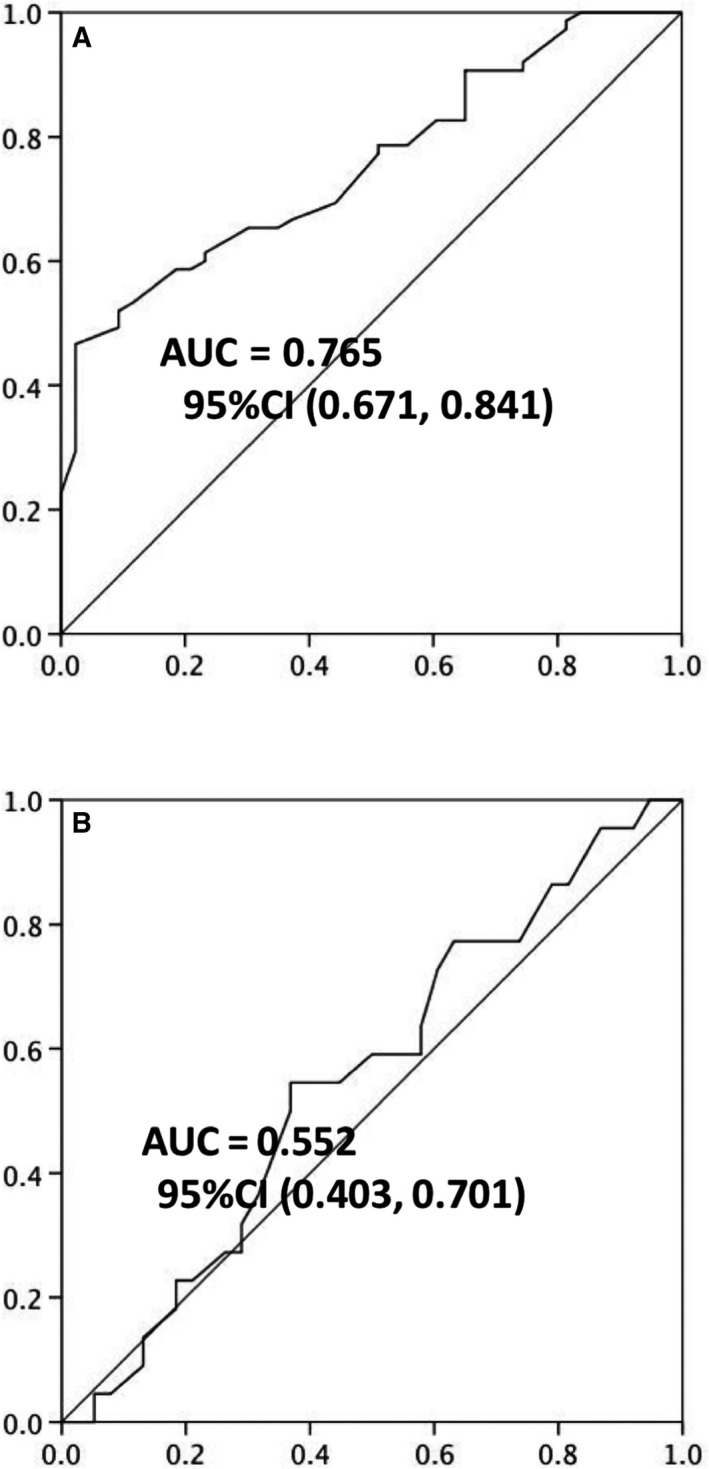
The receiver operator characteristic (ROC) curve depicted according to the diagnostic test evaluation revealed the prediction value of TSLP levels in the patients with sepsis with HL and higher ratio of neutrophils. (A) The ROC curve of serum levels of TSLP in prediction of mortality in the patients with HL and higher ratio of neutrophils. (B) The ROC curve of serum levels of TSLP in prediction of mortality in the patients without HL and higher ratio of neutrophils. The AUC and 95% confidence interval (CI) were presented in the figures.

We performed the diagnostic test evaluation to evaluate whether TSLP levels predict mortality in patients with sepsis. We found the cutoff value according to the diagnostic test evaluation to evaluate mortality risk between patients with or without HL and HNR. For mortality prediction, the cutoff value was 350 pg·mL^−1^ in patients with HL and HNR and 327 pg·mL^−1^ in patients without HL and high ratio of neutrophils. The AUC of the corresponding cutoff values was 0.85 and 0.71, respectively. The sensitivity was 89% and 62% and the specificity was 79% and 68% in patients with or without HL and HNR, respectively. The diagnostic efficiency of TSLP levels was significantly better in patients with higher absolute number of leukocytes and elevated ratio of circulating neutrophils (Fig. 6). Therefore, TSLP may serve as a potential biomarker for the evaluation of the mortality risk in patients with sepsis with HNR and HL.

## Discussion

The protein TSLP is a proinflammatory cytokine derived from epithelial tissues involved in inflammatory diseases through its binding to TSLPR. The TSLP/TSLPR signaling results in the activation of various innate and adaptive immune cells, including DCs, Treg, Th2 and Th1, indicating the complex influence of TSLP on inflammation 12, 13. Previous studies have focused on the effect of TSLP on allergic diseases, especially asthma 14. These studies have found that TSLP promotes Th2 differentiation through the activation of IL‐4 gene transcription, which aggravates type II inflammation. However, the effects of TSLP on infectious inflammation are unclear.

The TSLPR is widely expressed on various cells, including neutrophil, the key cell type involved in the killing of bacteria and control of acute infection 15, 16, 17, 18. Sepsis is characterized by toxin release after severe bacterial infection or endotoxemia caused by gram‐negative bacterial infection, leading to SIRS and subsequent multiple organ dysfunction 10, 19. The therapeutic intervention for the control of bacterial infection is of prime importance; therefore, aside from the administration of antibiotics, the function of neutrophils may be useful to evaluate the clinical outcome in patients with sepsis, especially in those with drug‐resistant bacterial infection, a leading cause of death in the ICU. In a recent study, TSLP increased the killing abilities of neutrophils in a skin infection mouse model induced by methicillin‐resistant *S. aureus* 20 *. *The increase in the neutrophil function is dependent on the complement activation, and this study revealed an unrecognized, non‐Th2‐associated, downstream signaling pathway of TSLP/TSLPR involved in the host defense and infectious inflammation. TSLP could enhance the antibacterial effect through the engagement of complement C5 and induction of excessive reactive oxygen species. This effect could result in a dual effect on sepsis‐induced systemic inflammation, including promotion of neutrophil function for the phagocytosis of drug‐resistant bacterial strains and aggravation in the production of proinflammatory cytokines and formation of inflammatory cascade. In the study on the antibacterial effect of TSLP, stimulation with HKSA enhanced the expression of TSLPR and reduced the number of colony‐forming units, but the neutrophils were derived from healthy controls, and the enhanced antibacterial function was likely associated with the activation of the toll‐like receptor‐2 signaling pathway. However, during the sepsis infection in humans, the innate immune signaling pathway is activated by a variety of pathogen‐associated molecular patterns and damage‐associated molecular patterns 10, 16. The infection induced by a single strain of *S. aureus* may incompletely simulate sepsis and may not meet the complexities involved in sepsis. Therefore, the TSLP‐induced antimicrobial effect dependent on neutrophils may fail to work in the case of other bacterial strains that participate in sepsis. In this study, we found that the function of neutrophils from patients with a higher level of TSLP have no discrimination when compared with the patients with a lower level. Neutrophils from patients were potentially exhausted and showed dysfunction. Neutrophils from patients with sepsis and severe systemic inflammatory syndrome were shown to be immature and less efficient in bacterial phagocytosis and production of reactive oxygen species 21. Furthermore, a novel subset of cultured neutrophils from patients with sepsis, CD16^high^CD24^high^, was shown to be significantly decreased because of the downregulation of the membrane CD24, and this effect was associated with the lack of neutrophil function in these patients, leading to an impairment in CD24‐mediated apoptosis of neutrophils and formation of a neutrophil extracellular trap 22. These studies demonstrated that the dysfunction of neutrophils may occur in patients with sepsis. Therefore, the antibacterial effects of TSLP attributed to the production of reactive oxygen species in neutrophils from healthy individuals observed in *in vitro* experiments may disappear because of the functional defects in neutrophils from patients with sepsis. On the contrary, the high TSLP level would activate the immune cells and boost the release of proinflammatory cytokines, which may enhance the proinflammatory phenotype and deteriorate the inflammatory cascade response.

Human TSLPR is shown to be preferentially coexpressed with IL‐7Rα on myeloid‐derived monocytes and DCs. TSLP could promote the differentiation of myeloid‐derived DCs and Th17 response in hepatitis C virus–infected hepatocytes 23, 24.

In sepsis, excessive systemic inflammation may lead to multiple organ dysfunction and worsen prognosis. TSLP serves as a classical proinflammatory cytokine that has potential effects on patients with sepsis with severe SIRS. In two previous animal studies, opposite effects of TSLP in septic mice were observed 8, 9. Kuethe et al. found that TSLP could improve the survival of septic mouse through the reduction in inflammation. In TSLPR^−/−^ mouse, an increase in mortality was observed after CLP. The expression of IL‐6 and IL‐12 in peritoneal myeloid cells was significantly increased in CLP‐induced Lys‐Cre^+^;tslp^fl/fl^ mouse as compared with Lys‐Cre^−^;tslp^fl/fl^ mouse. The lack of TSLP/TSLPR may result in the dysregulation of inflammation in terms of the release of cytokines and proinflammatory mediators. Another study showed that the administration of the neutralizing antibody to TSLP may improve survival and facilitate the killing of bacteria in a septic mouse, as observed with an increase in the level of peritoneal TSLP. This study suggests that anti‐TSLP failed to inhibit neutrophil infiltration or induce normal production of proinflammatory cytokines to control infection in the septic mouse.

Our study revealed the correlation between the levels of TSLP and prognosis in patients with sepsis. We found that the nonclassical and intermediate subsets of monocytes from patients with high levels of TSLP showed the characteristic proinflammatory phenotype. The two subsets of monocytes were preferentially mobilized during inflammation and expanded in response to macrophage colony‐stimulating factor. In comparison with CD16^+^ monocytes, CD14^2+^CD16^−^ monocytes are primary myeloid‐derived cells associated with chemokine receptors such as CCR2 and CD62L 25, 26, 27. We found that patients with high levels of TSLP showed stimulated production of intermediate and nonclassical subsets of monocytes. CD4^+^ T cells from patients with high levels of TSLP similarly produced more proinflammatory cytokines but less anti‐inflammatory cytokines such as IL‐10. However, it has been proved that TSLP could aggravate phagocytosis and reactive oxygen species–producing abilities of neutrophils in previous studies 10, 18, 19, 20. In this study, we unexpectedly found that TSLP in patients with sepsis plays a proinflammatory effect in monocytes and lymphocytes but has no effect on the antibacterial activity of neutrophils, which indicated that TSLP could not enhance the antibacterial effect as found in previous animal studies and induced only pathological uncontrolled secondary inflammation response in patients with sepsis.

In this study, we hypothesized that higher levels of TSLP might result in poor prognosis, and explored the correlation between prognosis and levels of TSLP. We found that in patients with HNR and HL, high levels of TSLP would result in poor prognosis. We identified the cutoff value for the prediction of mortality and verified the cutoff value in patients. The cutoff value was higher in patients with HL and HNR, consistent with the aforementioned result that high TSLP level showed positive correlation with poor prognosis only in patients with HL and HNR.

This study also has some limitations. First, considering that this study is only a preliminary observational human study, the further mechanisms involved in the influence of TSLP on severe bacterial infection and sepsis. Although we found the circulating myeloid‐derived cells from patients with a higher level of TSLP have more significant proinflammatory phenotype, the underlying mechanisms need to be investigated in further animal experiments and basic research. Second, although our data about neutrophils cannot completely illustrate and interpret these functions of neutrophils, further evaluation about the neutrophils in the patients with higher TSLP is necessary in the future. Third, the sample size in the diagnosis evaluation test is relatively small. More reliable conclusions should be drawn in a trial of a larger, prospectively validated disease cohort.

## Conclusions

Our study provides a potential biomarker for the prediction of prognosis in a subgroup of patients with sepsis. We revealed the inflamed phenotype of immune cells in patients with sepsis with high levels of TSLP. We propose that the cutoff value of serum levels of TSLP could be used for risk assessment in the patients with sepsis with HNR and HL according to diagnostic test evaluation and may have potential value in the management of sepsis.

## Conflict of interest

The authors declare no conflict of interest.

## Author contributions

QY and HX conceived the study and designed the experiments. YL and HW contributed to the data collection, performed the data analysis and interpreted the results. QY wrote the manuscript. HX contributed to the critical revision of this article. All authors read and approved the final manuscript.
