# Corrigendum to: TSLP induces a proinflammatory phenotype in circulating innate cells and predicts prognosis in sepsis patients

**DOI:** 10.1002/2211-5463.13739

**Published:** 2023-12-20

Qichuan Yu, Yang Li, Hao Wang, Huawei Xiong (2019) TSLP induces a proinflammatory phenotype in circulating innate cells and predicts prognosis in sepsis patients. *FEBS Open Bio* 9: 2137–2148. https://doi.org/10.1002/2211‐5463.12746

In the above article, the co‐author's name Qichuan Yu was spelt incorrectly. The correct spelling is Qichun Yu. The co‐author's name has been corrected in the original article.

